# Follow-Up Recommendations after Curative Resection of Well-Differentiated Neuroendocrine Tumours: Review of Current Evidence and Clinical Practice

**DOI:** 10.3390/jcm8101630

**Published:** 2019-10-05

**Authors:** Angela Lamarca, Hamish Clouston, Jorge Barriuso, Mairéad G McNamara, Melissa Frizziero, Was Mansoor, Richard A Hubner, Prakash Manoharan, Sarah O’Dwyer, Juan W Valle

**Affiliations:** 1 Medical Oncology Department, The Christie NHS Foundation Trust, Manchester M20 4BX, UK; Jorge.Barriuso@manchester.ac.uk (J.B.); mairead.mcnamara@christie.nhs.uk (M.G.M.); Melissa.Frizziero@christie.nhs.uk (M.F.); Was.Mansoor@christie.nhs.uk (W.M.); Richard.Hubner@christie.nhs.uk (R.A.H.); 2 Division of Cancer Sciences, University of Manchester, Manchester M13 9PL, UK; Sarah.O'Dwyer@christie.nhs.uk; 3 Surgery Department, Colorectal and Peritoneal Oncology Centre, The Christe NHS Foundation Trust, Manchester M20 4BX, UK; Hamish.Clouston@christie.nhs.uk; 4 Radiology and Nuclear Medicine Department, The Christie NHS Foundation Trust, Manchester M20 4BX, UK; Prakash.Manoharan@christie.nhs.uk

**Keywords:** neuroendocrine tumours, neuroendocrine neoplasms, curative surgery, resection, follow-up, guidelines, relapse, recurrence, risk factor

## Abstract

The incidence of neuroendocrine neoplasms (NENs) is increasing, especially for patients with early stages and grade 1 tumours. Current evidence also shows increased prevalence, probably reflecting earlier stage diagnosis and improvement of treatment options. Definition of adequate postsurgical follow-up for NENs is a current challenge. There are limited guidelines, and heterogeneity in adherence to those available is notable. Unfortunately, the population of patients at greatest risk of recurrence has not been defined clearly. Some studies support that for patients with pancreatic neuroendocrine tumours (PanNETs), factors such as primary tumour (T), stage, grade (Ki-67), tumour size, and lymph node metastases (N) are of relevance. For bronchial neuroendocrine tumours (LungNETs) and small intestinal neuroendocrine tumours (siNETs), similar factors have been identified. This review summarises the evidence supporting the rationale behind follow-up after curative resection in well-differentiated PanNETs, siNETs, and LungNETS. Published evidence informing relapse rate, disease-free survival, and relapse patterns are discussed, together with an overview of current guidelines informing postsurgical investigations and duration of follow-up.

## 1. Introduction

Neuroendocrine neoplasms (NENs) are rare and heterogeneous [1,2]. Assessment of stage, primary tumour site, and tumour grade are the cornerstones for treatment planning [3,4]. 

For gastro-entero-pancreatic (GEP)-NENs, the World Health Organisation (WHO) tumour grade is defined by the percentage of tumour cells with a nuclear expression of Ki-67 and morphological differentiation features (well-differentiated (called neuroendocrine tumour (NET)) vs. poorly-differentiated (called neuroendocrine carcinoma (NEC))) [5,6,7]. Following these criteria, GEP-NENs are classified as follows: grade (G) 1-NET (Ki-67 < 3%; well-differentiated morphology), G2-NET (Ki-67 3–20%; well-differentiated morphology), G3-NET (Ki-67 > 20%; well-differentiated morphology), and G3-NEC (Ki-67 > 20%; poorly-differentiated morphology). Lung-NENs are divided according to morphology into lung carcinoids (well-differentiated morphology) and lung NECs (poorly-differentiated morphology). Lung carcinoids are subdivided into Typical Lung Carcinoid (defined as <2 mitosis per 10 high-power fields (HPF) and absence of necrosis) and Atypical Lung Carcinoid (defined as 2–10 mitosis per 10 HPF and focal necrosis). Lung NECs are characterised by >10 mitosis per 10 HPF and diffuse necrosis and can be subdivided into large and small cell lung NECs according to cell morphology [8,9]. The role of Ki-67 in lung NETs has not been validated [10]. 

For patients with localised disease, surgery is the treatment of choice, especially for G1 and G2 NETs [11,12,13]. There is no clear evidence supporting adjuvant treatment for resected NETs [11,13], and scarce retrospective evidence available, mainly focused on lung NECs and high risk PanNETs [14,15]. Advanced disease is only amenable to palliative treatment with the aim of prolonging overall survival [13,16]. 

While multiple clinical trials have explored the most suitable treatment strategies for patients with advanced disease [16], the optimal postsurgical follow-up for patients with resected NENs remains unclear, with no prospective clinical trials in this setting, and variable adherence to current guidelines [17]. The definition of adequate post-resection follow-up for NENs is one of the challenges currently faced by both individual clinicians and multidisciplinary teams. 

This review summarises the available evidence supporting follow-up after curative resection of sporadic (nonhereditary) well-differentiated NETs arising from the pancreas (PanNETs), small intestine (excluding appendix) (siNETs), and lung (LungNETS). This manuscript also reviews current guidelines and identifies areas of uncertainty to be addressed by future research. Since surgery for poorly-differentiated tumours has a limited role, the focus of these recommendations will be limited to patients with well-differentiated tumours. In addition, this manuscript will not cover specific recommendations for NETs arising from the appendix or rectum.

## 2. How Large Is the “Resected” Population?

Both the incidence and prevalence of patients with localised NETs is gradually increasing, and consequently [1], the amount of patients with resected NETs who would meet criteria for postsurgical follow-up is also increasing. There is therefore a clear need to define the best follow-up following resection. The latest evidence from the Surveillance, Epidemiology, and End Results (SEER) programme analysed 64,971 cases of NENs, and confirmed an increase in incidence with an age-adjusted annual incidence rate increase by 6.4-fold from 1973 (1.09 per 100,000) to 2012 (6.98 per 100,000) [1]. This increase occurred across all sites, stages, and grades. The highest incidences were recorded for Lung NENs (1.49 per 100,000), followed by GEP NENs (3.56 per 100,000), and NENs from an unknown primary (0.84 per 100,000). In addition, the highest incidence increase was recorded for localised stage disease (from 0.21 per 100,000 persons in 1973 to 3.15 per 100,000 persons in 2012; *p* value < 0.001) and G1-NETs (increased from 0.01 per 100,000 persons in 1973 to 2.53 per 100,000 persons in 2012; *p* value < 0 .001) [1]. Whether this is a true incidence increase or an improved recognition and diagnosis remains unclear.

The median overall survival (OS) for all NENs (regardless of primary site, stage at diagnosis, or grade) was 9.3 years. As expected, longer OS (median >30 years) was reported for patients with localised (resectable) NENs when compared to those with regional (locally advanced) (median OS 10.2 years) and distant (metastatic) NENs (median OS 12 months) (*p* value < 0 .001) [1]. Taking into account that 52.3% (28,031 out of 53,565) of the population presented with localised disease, and the prolonged OS in this population, the 20 year limited-duration prevalence also increased, from 0.006% in 1993 to 0.048% in 2012 (*p* value < 0.001).

## 3. There Is a Need to Standardise Current Practice

Even though guidelines for the postsurgical resection of PanNETs, siNETs, and LungNETS [11,18,19,20,21] are available, a recent study by Chan et al. showed that these are far from being widely adopted by clinicians, and that practice is heterogeneous [17]. Published in 2018, this practice survey of the Commonwealth Neuroendocrine Tumour Collaboration (CommNETS) and the North American Neuroendocrine Tumor Society (NANETS) gathered information regarding follow-up patterns by health care practitioners and identified areas of variation in practice [17]. A total of 163 responses to a web-based survey targeting NET health care providers in Australia, New Zealand, Canada, and the United States were received. Responding specialties included 50% medical oncology, 23% surgery, and 13% nuclear medicine (with 15% other). A large proportion of responders confirmed they were aware of follow-up guidelines, such as those from the National Comprehensive Cancer Network (NCCN) (38%), the European Neuroendocrine Tumor Society (ENETS)) (33%), and the European Society for Medical Oncology (ESMO) (17%). In contrast, only 15%, 27%, and 10%, respectively, found these guidelines “very useful”, and 63% reported not to use them. Responders agreed that grade, followed by Ki-67, was the most relevant prognostic factor in the population of patients with resected NENs, while the site of origin was not felt to be of much relevance. Around half of responders reported that they followed-up resected patients for longer than 5 years (26% 6–10 years; 23% >10 years). The frequency at which such follow-up was performed varied across responders (the majority performed 3–6 monthly visits during the first 1–2 years, followed by annual visits thereafter), as did the investigations carried out at follow-up (serum tumour markers (Chromogranin A (CgA) 86%) and computerised tomography (CT 66%) were the more frequently employed tests). Only 40% of responders performed radiological assessment after the first 5 years of follow-up post-resection [17]. No other studies exploring this issue are available to date.

These results highlight the huge variability in practice and in adherence to current guidelines. One of the reasons for such poor adoption may be the lack of high quality evidence behind the available recommendations, together with the lack of evidence to favour specific follow-up tools over others. This variability in follow-up can explain not only the lack of quality retrospective data (unreliable in view of different follow-up strategies adopted across countries/centres), but also the challenges in identifying populations at increased risk of relapse, which may enable prospective development of adjuvant strategies. 

It is therefore of major importance to work towards an improved standardisation of follow-up for patients with resected NETs. 

## 4. Why? Rationale for Follow-Up

Current guidelines are available with post curative resection follow-up recommendations for all patients diagnosed with PanNETs [11,18,19,22,23], siNETs [18,21,22,23], and LungNETs [11,18,22,23]. 

The main factor supporting long-term postsurgical follow-up is not only the risk of recurrence, but the risk of late recurrence. The duration of post curative resection follow-up seems to be associated with the risk of later recurrence and recommendations are individualised for each cancer subtype. The risk of breast cancer recurrence continues through 15 years after primary treatment and beyond, and based on this, long-term mammography is recommended [24]. In contrast, for lung and colorectal cancer, most recurrences will occur within the first 2 years (maximum of 5 years) from the time of curative surgery, and current guidelines recommend follow-up for up to 5 years after curative resection [25,26].

It is anticipated that through regular follow-up, relapse should be diagnosed earlier, when surgical strategies with curative intent may be of benefit [27]. Benefits of postsurgical follow-up have shown to translate into significant improvements in patient overall survival in patients with breast cancer [28]. Evidence in colorectal cancer varies between studies, with some studies suggesting a benefit in terms of overall survival [26], while others showed a minimal impact on survival benefit despite higher rate of resectable disease being identified [29]. Such a benefit has not been shown in NENS, and prospective studies should be performed in this setting.

### 4.1. Resected PanNETs 

Multiple retrospective series have explored the risk of relapse following surgery for PanNETs. Some of the most recent series are summarised in Table 1 [30,31,32,33,34]. Risk of tumour recurrence varied between series (12%–25%–69%) [30,34,35], as did the reported disease-free survival (19–55 months) [30,33]. In the series published by Sho and colleagues, patients diagnosed with PanNETs who underwent surgical resection between 1989 and 2015 were reported [31]. Of the 140 patients included, relapse-free survival dropped significantly after 5 years of follow-up (5 and 10 year relapse-free survival was 84.6% and 67.1%, respectively). It is also worth noting that some series have reported shorter disease-free survivals for PanNETs (vs. other NETs). In a series reporting data of over 900 patients with NETs, the median DFS among patients with resected siNET or PanNETs was 5.8 and 4.1 years, respectively [36]. Similar trends were reported by Singh et al. [35]. Such findings, together with identification of tumour recurrence after 10 years of follow-up [34,35], suggest that long-term follow-up is required for PanNETs. Some of the series did highlight an incongruence between the pattern of follow-up imaging performed (more frequent assessment during the first 3 years postsurgery) and the time-to-recurrence reported (only one third of patients recurred over this period of time. Cumulative incidence of recurrence was 26.5%, 39.6%, 57.0%, and 69.4% at 3, 5, 10, and 15 years post-resection, respectively [35]. 

The site of tumour recurrence has been described to be predominantly distant, with a tropism for liver metastases [31,34,37]. Rates of local recurrence are variable between series [32]. There seems to be an increased rate of pancreas-only recurrence associated with surgical margin status [38].

### 4.2. Resected siNETs 

Despite expected indolent clinical behaviour, the risk of relapse reported seems to vary according to the length of follow-up between studies [35,36]. In a series of 936 patients (of whom 43 were siNETs), the cumulative incidence of recurrence for the siNET population was 22.8%, 33.8%, 52.9%, and 62.0% at 3, 5, 10 and 15 years post-resection, respectively [35]. In view of the risk of relapse even a long time after resection, long-term follow-up is recommended. As previously mentioned, disease-free survival seems to be longer for siNETs than for PanNETs, with median disease-free survivals varying among studies [35,36]. 

The relapse sites have been reported to be distant, with liver predominance [36]. 

### 4.3. Resected LungNETs

Some retrospective series have explored the risk of recurrence following resection of LungNETs. The series by Lou and colleagues reported a 6% recurrence rate after a median follow-up time of 3.5 years within a population of 337 patients with resected LungNETs. Sites of recurrence were mainly distant, with predominance of liver and bone metastases [39]. Of the 21 patients with tumour recurrence, only one had evidence of local relapse. Whether longer time of follow-up would impact the relapse rate reported remains unclear. Current guidelines recommend long-term follow-up in view of risk of late relapse (up to 19% of relapses were 7 years from resection in some series) reported in the literature [40,41].

## 5. For Whom? Risk Stratification

As specified above, and in view of the risk of tumour recurrence, current guidelines recommend follow-up for all resected patients with PanNETs, siNETs, and LungNETs [11,18,19,20,21]. However, recommendations from current guidelines provide few insights regarding individualised recommendations based on individual tumour characteristics, which may derive an increased relapse risk. Thus, risk stratification may be of relevance not only to reduce exposure to radiation in these patients with lower risk, but also to ensure adequate use of resources and to identify target populations for future clinical trials in the adjuvant setting. Some recent publications provide initial recommendations that tailor the frequency of follow-up investigations to tumour characteristics such as size, Ki-67, or lymph node metastases, but these are not yet adopted by international guidelines [42,43,44]. Development of molecular markers for risk of recurrence stratification are under development, but remain investigational and not available for its use in daily clinical practice [45].

### 5.1. Resected PanNETs 

Reported series are consistent regarding the increased risk of relapse related to tumour size (>2 cm), presence of lymph node metastases in the resection specimen (N+), grade 2 tumours (vs. grade 1), and the presence of involved microscopic resection margins (R1) (vs. clear resection margins (R0)) [30,31,32,33,34,44]. Other factors such as Ki-67 above 5%, tumour functionality, and the presence of perineural and vascular invasion have also been suggested as factors related to increased relapse risk. However, these observations are not consistent between series and are likely to require further validation [30,32,34,46]. It is considered that resected insulinomas with N0 and R0 disease are the PanNETs with the lowest risk of tumour recurrence [19]. There has been recent evidence supporting the impact not only of presence/absence of affected lymph nodes, but also the number of these. Partelli and colleagues showed that the presence of 4 or more lymph node metastases (N2 disease) correlated with a lower 3 year disease-free survival (75%) when compared with presence of 1-3 positive lymph nodes (N1 disease; 3 year disease free survival rate of 83%) and N0 disease (3 year disease free survival rate of 89%) [47]. Authors also suggested that a minimum of 13 examined lymph nodes seemed to be adequate for such assessment [47]. 

### 5.2. Resected siNETs 

One of the most relevant risk factors to predict increased relapse risk in patients with siNETs is the presence of lymph node metastases in the resected specimen. Zaidi and colleagues reported a series of 199 patients with resected siNETs [48]. Of the whole population, 154 patients (77.4%) had lymph node-positive disease. No difference in 3 year recurrence-free survival was found between patients with lymph node-positive (N+) and lymph node-negative (N0) disease. However, authors demonstrated that patients with four or more positive lymph nodes had a worse 3 year recurrence-free survival (81.6% vs. 1–3 (91.4%) or 0 (92.1%) lymph nodes affected; *p* value 0.01). In addition, the authors also concluded that retrieval of eight or more lymph nodes at time of surgery was required to accurately evaluate number of lymph nodes involved. Other risk factors such as grade (grade 2; especially if Ki-67 > 10%) and T3/4 tumours have also been reported [35,36].

### 5.3. Resected LungNETs

Risk factors associated with increased relapse risk for LungNETs include the presence of positive lymph nodes (N+) [39,49] and the presence of atypical LungNETs (26%; vs. 3% in typical LungNETs) for whom time to recurrence was also shorter (median 1.8 years (range 0.2–7 years) vs. 4 years (range 0.8–12 years) for typical LungNETs) [39]. Other series have also reported that mitotic index, Ki-67 index, and the presence of necrosis were independent prognostic factors for relapse-free survival in resected LungNETs [10]. 

## 6. How? Presurgical Staging and Follow-Up Tools

In addition to patient history and physical examination, cross-sectional imaging (both in the form of CT and magnetic resonance imaging (MRI)) is one of the main tools for patient follow-up. Reduction of exposure to radiation is to be considered when planning for long-term follow-up, especially in young patients, and alternating CT and MRI (or limiting to MRI alone) could be an alternative in selected scenarios [50]. In addition, use of serum/urine biomarkers and nuclear medicine imaging have a role that warrants further discussion [23]. Table 2 provides a summary of the recommendations for each one of the assessments discussed in this section [11,18,19,20,21,22,23], both for baseline assessment and follow-up. Table 3 provides a summary of further recommendations, including timing and frequency of examinations suggested by current guidelines [11,18,19,20,21,22,23].

### 6.1. Currently-Available Biomarkers

Histologically, NETs share common features, such as specific secretory granules often containing biogenic amines and polypeptide hormones that help with the diagnostic process. Chromogranin and synaptophysin are examples of tumour markers utilised, while neuron-specific enolase (NSE) is less specific [51]. The use of staining with peptide hormones, such as insulin, glucagon, or other specific peptides is of use in selected cases only, when such diagnoses as insulinoma, glucagonoma, etc. are suspected [52].

Serum/urine markers can be useful not only for diagnosis in patients with NETs, but also for follow-up. Serum markers of relevance include Chromogranin A (CgA), which is co-secreted with other hormones by neuroendocrine tumour cells [52]. Only 10%–40% of PanNETs are functioning tumours [19]. Based on this, measurement of serum pancreatic polypeptide, insulin, glucagon, somatostatin, gastrin, vasoactive intestinal polypeptide (VIP), and others has a role if patients’ symptoms suggest a particular diagnosis related to these secretions [19]. In patients with siNETs, quantification of 5-hydroxyindoleacetic acid (5-HIAA), a metabolite of serotonin, in either serum or urine is recommended [53]. The use of serum NSE is usually limited to poorly-differentiated NECs [54], with some evidence suggesting its role for atypical LungNETs [55].

In the setting of localised resectable disease, baseline assessment (preferably presurgery) of the above-mentioned serum/urine tumour markers could inform which the most suitable serum marker for follow-up after surgery on an individual patient basis is [52]. However, clinicians should bear in mind potential false positive findings when performing biochemistry follow-up [56]. Discrepancy between guidelines exists in this setting, and while most guidelines support the role of biochemistry follow-up for patients with resected NETs [11,18,19,20,21,23], the CommNETs/NANETS guidelines do not fully support such an approach outside the scenario of patients with functioning PanNETs [22]. 

While other novel biomarkers are currently being developed, their use has not been validated in this setting [57,58,59].

### 6.2. The Evolving Role of Nuclear Medicine

One of the suggested reasons for the increasing incidence of NETs is the improvement in diagnostic imaging techniques [60]. ^18^Fluoro-deoxyglucose (^18^F-FDG), was one of the first tracers developed in oncology. However, its role in the diagnosis and/or follow-up of NENs is considered more relevant in patients with poorly-differentiated NENs [13,61,62,63,64,65].

The expression of somatostatin receptors (SSTR) on the cell membrane is one of the unique characteristics of NETs, which makes SSTRs a suitable molecular target for specific diagnostic and therapeutic ligands. Based on this, the nuclear medicine field has targeted SSTRs for diagnosis and treatment of NETs for decades [66]. The vast majority of research has been focused on Indium-111 (^111^In)-pentetreotide (Octreoscan^®^), which was the only approved agent for the scintigraphic localisation of primary and metastatic NETs [67]. More recently, the clinical use of gallium-68 (^68^Ga)-labelled compounds has increased in NETs [68,69] due to its affinity for multiple SSTR subtypes (SSTR2, SSTR3, SSTR5) [70]. In addition, the first Ready-to-Use (SOMAKIT TOC®) ^68^Ga-DOTA0-Tyr3-Octreotide (^68^Ga-DOTATOC) for injection was approved for use in patients diagnosed with GEP-NETs [71].

In the last decade, several clinical studies have compared the diagnostic role of ^68^Ga-DOTA-PET to somatostatin receptor scintigraphy (Octreoscan^®^) in patients diagnosed with NETs. These studies have confirmed an increased sensitivity and image quality, together with an increased capacity to detect additional lesions and alter management in favour of ^68^Ga-DOTA-PET [72,73,74,75,76,77,78]. 

A recent systematic review and meta-analysis of 22 studies exploring the role of ^68^Ga-DOTA-PET in NETs reported a high pooled sensitivity (93% (95% CI 91%–94%)) and specificity (96% (95% CI 95%–98%)) [79]. The only exceptions to this high performance are insulinomas, for which lower sensitivities have been reported [80]. In addition, the use of ^68^Ga-DOTA-PET in PanNETs has been reported to provide useful additional information, and impacted on patient management, in 20%–55% of cases [81,82,83,84]. 

For LungNETs, studies have reported a more selective uptake of ^68^Ga-DOTA for typical LungNETs, while atypical LungNETs demonstrated less ^68^Ga-DOTA uptake and increased ^18^F-FDG avidity [85]. 

A retrospective series of 46 patients with LungNETs assessed with ^68^Ga-DOTA-PET reported that the ^68^Ga-DOTA-PET provided additional information in 37% of patients and impacted on management in 26% [78]. A change in management was due to identification of occult sites in nine patients, three of whom were patients in the postsurgical setting. No differences in the rate of practice-changing ^68^Ga-DOTA-PET results by type of LungNET were reported in this series.

Based on the above evidence, SSTR imaging (preferably in the form of ^68^Ga-DOTA-PET, if available) is the method of choice to fully stage patients with PanNETs, siNETs and LungNETs [19,21]. Such examination is recommended as a baseline assessment (preferably presurgery) or as a baseline postoperative assessment only (preferably after 3–6 months from surgery to avoid false positive findings [86]). Current guidelines do not recommend the use of nuclear medicine imaging for routine surveillance [11,18,19,20,21], with the exception of the ENETS guidelines which suggest consideration of SSTR imaging 2 yearly following resection for patients with known positive SSTRs before surgery [23]. Once again, possibility of false positive findings may challenge interpretation of results and should be taken into account by treating clinicians [87].

## 7. Summary of Current Guidelines

Current guidelines recommend post curative resection follow-up for all patients diagnosed with PanNETs [11,18,19,22,23], siNETs [18,21,22,23], and LungNETs [11,18,22,23]. Table 3 provides a summary of recommendations from current ENETS, NCCN, and CommNETs/NANETS guidelines [11,18,19,20,21,22,23]. Recommendations regarding who the most appropriate specialist is to perform such follow-up do not exist.

Even though most guidelines available agree regarding the type of investigations to be performed, the frequency of such examinations varies significantly between them. In addition, follow-up beyond 10 years is suggested as a discussion point to have with patients on an individual patient basis by the NCCN and CommNETs/NANETS guidelines, while the ENETS guidelines suggest life-long follow-up for well-differentiated NETs. Finally, biochemistry follow-up is not strongly recommended by the CommNETs/NANETS guidelines, in view of the limited impact on patient management, with the exception of functional PanNETs [22]. ENETS guidelines also suggest the role of regular postsurgical SSTR imaging in patients with known previous uptake pre-resection [23]. 

## 8. Conclusions and Future Steps

The available literature confirms that following resection of well-differentiated NETs, and despite their “indolent” behaviour, relapse rate can be frequent, especially if long-term follow-up is adopted [35]. Reported relapse rates vary between series, probably due to the variability regarding follow-up recommendations between available guidelines, together with lack of adherence to such guidelines, not only between countries but also between centres in the same country [17]. Some of the main discrepancies between the available guidelines are related to the frequency of assessments, the role of biochemistry follow-up, and the role of SSTR imaging. Unless prospective studies are pursued to clarify the real benefit and impact of such investigations on patients’ outcome, these will remain unclear with continued practice variability. The risk/benefit ratio of degree of exposure to radiation does also require to be taken into account. The development of novel biomarkers should be exposed to scrutiny with mandated validation before being adopted into guidelines.

In view of the increasing incidence of NETs, mainly in the form of localised stages, standardisation of follow-up strategies with the potential to increase cure rate is becoming an urgent need for the field to move forward. In order to achieve such an impact, development of adjuvant strategies in NETs will need to be explored. Few studies have explored this and there are many associated challenges. Firstly, NETs are rare tumours, and when focusing on a resectable patient population potentially eligible for clinical trials, recruitment may be a barrier, unless studies are designed within international networks. Secondly, the heterogeneity of NETs would make it mandatory for trials to focus on specific patient populations (PanNETs, siNETs, or LungNETs) which would be an additional challenge to recruitment. Thirdly, and in view of long-term relapse patterns, such clinical trials would require long-term follow-up with the associated cost. A potential solution for this would be to focus on patients with an increased risk of tumour recurrence, allowing for stratification based on the available retrospective evidence. Finally, based on the high rate of distant relapse, systemic treatment would need to be explored, if an adjuvant study was to be designed.

In summary, current guidelines and clinical practice vary regarding follow-up recommendations in patients with NETs. Standardised practice and agreement between guidelines is required to secure homogeneity of follow-up, better identification of patients at risk of recurrence, and an adequate study design exploring adjuvant strategies in patients with NETs to increase the cure rate statistics. Prospective studies performed in this setting, together with high quality patient registries, are required to move the field forward.

## Figures and Tables

**Table 1:** Most relevant retrospective series in PanNETs.

Author; Year	Relapse Rate	Risk Factors	Site of Recurrence
**Gao et al. 2018** [30]	Relapse rate 129/505 (25.5%). Median disease-free survival of 19 months (range 6–96 months).	T3, T4, N+, Ki-67 >2%, functional	Not reported
**Sho et al. 2018** [31]	Relapse rate 23/140 (16.3%). 5 and 10 year relapse-free survival was 84.6% and 67.1%, respectively.	Size >5 cm, N+, Ki-67 >20%	All recurrence was distant (liver, peritoneal, and bone)
**Genç et al. 2018** [32]	Relapse rate 35/211 (17%). The 5 and 10 year disease-specific/overall survival was 98%/91% and 84%/68%, respectively. Median time to recurrence was 43 months (IQR 23–62).	Grade 2, N+, perineural invasion	Pancreatic remnant (69%), distant (14%), 1 patients had lymph node metastasis
**Ausania et al. 2019** [33]	Relapse rate 19/137 (13.9%). Median DFS was 55 months.	Tumour size >2 cm, N+, Ki-67>5% or mitotic index >2	Not reported
**Marchegiani et al. 2018** [34]	Relapse rate (12.3%) Recurrence occurred either during the first year of follow-up (*n* = 9), or after ten years (*n* = 4).	>21 mm size, G3, N+, vascular infiltration	Liver (11.1%), local recurrence (2.3%), lymph node (2.1%), other organs (1.6%)
**Singh et al. 2018** [35]	Cumulative incidence of recurrence was 26.5%, 39.6%, 57.0%, and 69.4% at 3, 5, 10 and 15 years post-resection, respectively.	Not reported	Not reported

Summary of the latest and largest retrospective series exploring relapse rate and risk of relapse for patients diagnosed with resected pancreatic neuroendocrine tumours (PanNETs) [30,31,32,33,34,35]. *n*, number; N, lymph node; N+, affected lymph node; T, primary tumour; IQR, interquartile range; DFS, disease-free survival; G, grade.

**Table 2:** Summary recommendations of baseline and follow-up tools in assessment of patients after curative resection of neuroendocrine tumours. Adapted from [11,18,19,20,21].

		Biochemistry				Cross-Sectional Imaging (CT/MRI)	SSTR Imaging (^68^Ga-DOTA-PET)	^18^F-FDG -PET
		CgA (Serum)	5-HIAA (Serum/Urine)	Pancreatic Peptides (Serum)	NSE			
**PanNETs**	**First assessment**	✓	×	If functional	×	✓	✓	×
	**Follow-up**	✓	×	If functional	×	✓	**	×
**siNETs**	**First-assessment**	✓	✓	×	×	✓	✓	×
	**Follow-up**	✓	If elevated at diagnosis	×	×	✓	**	×
**LungNETs**	**First assessment**	✓	✓	×	If atypical	✓	✓	If atypical
	**Follow-up**	✓	If elevated at diagnosis	×	If elevated at diagnosis	✓	**	#

PanNETs, well-differentiated pancreatic neuroendocrine tumours; siNETs, well-differentiated small intestinal neuroendocrine tumours; LungNETs, well-differentiated lung carcinoids (typical/atypical); CgA, chromogranin A; 5-HIAA, 5-hydroxyindoleacetic acid; NSE, neuron-specific enolase; CT, computerised tomography; MRI, magnetic resonance imaging; SSTR, somatostatin receptor; PET, positron-emission tomography; ^18^F-FDG, fluorodeoxyglucose; ^68^Ga-DOTA, 68Ga-dodecanetetraacetic acid. ** According to the ENETS guidelines, follow-up with SSTR imaging if positive at diagnosis could be considered. # According to the ENETS guidelines, follow-up with ^18^F-FDG PET, if positive at diagnosis could be considered for atypical LungNETs.; ✓ Recommended; × Not recommended.

**Table 3:** Summary of patient outcomes and follow-up recommendations. Outcome data extracted from [30,31,32,33,34,35]. Recommendations adapted from [11,18,19,20,21,22,23].

	Relapse Rate	Late Relapse	Site of Recurrence / Metastases	Risk Factors for Relapse	Staging	Follow-up Recommendations (ENETS, NCCN, CommNETs/NANETS		
					Pre-Surgery	3–12 Month Post-Resection	After 1st Year and Until 10 Years Post-Resection	After 10 Years
**PanNETs**	12%–25% up to 70% at 15 years of follow-up	>5–10 years	Liver > local	Size/T, N, Ki-67/grade	Cross-sectional imaging (CT/MRI) + SSTR imaging + CgA (+ pancreatic peptides if functioning)	History and physical examination + biochemistry * + cross-sectional imaging (CT/MRI)	Frequency: 3–6 monthly for nonfunctional PanNETs and 6–12 monthly for functional PanNETs (ENETS) / 6–12 monthly (NCCN) / every year for 3 years, every 1–2 years thereafter (CommNETs/NANETS). As per ENETS guidelines insulinomas may not require any radiological follow-up Examinations: History and physical examination + biochemistry * + cross-sectional imaging (CT/MRI) ENETS guidelines suggest 1–2 yearly SSTR imaging if positive at diagnosis	Individualised decision to continue; recommended life-long (ENETS)
**siNETs**	20%–50%; up to 62% at 15 years of follow-up	10–15 years	Liver	Resected number of lymph nodes (>8) and positivity of those lymph nodes (>4)	Cross-sectional imaging (CT/MRI) + SSTR imaging + CgA (+ 5-HIAA)	History and physical examination + biochemistry * + cross-sectional imaging (CT/MRI)	Frequency: 6–12 monthly (ENETS) / every 1–2 years (CommNETs/NANETS/NCCN). Examinations: History and physical examination + biochemistry * + cross-sectional imaging (CT/MRI) ENETS guidelines suggest 2 yearly SSTR imaging if positive at diagnosis	Individualised decision to continue; recommended life-long (ENETS)
**LungNETs**	3%–26% depending on subtype	>7 years; atypical developed earlier relapse	Liver and bone	Mitotic index, Ki-67, necrosis, atypical, N	Cross-sectional imaging (CT/MRI) + SSTR imaging [18F-FDG-PET could be considered if atypical] + CgA (+ 5-HIAA + NSE [if atypical])		Frequency: 6–12 monthly (ENETS/NCCN); 3–6 monthly if atypical (ENETS). Examinations: History and physical examination + biochemistry * + cross-sectional imaging (CT/MRI) ENETS guidelines suggest 1–3 yearly SSTR imaging if positive at diagnosis (1–2 yearly for atypical); ^18^F-FDG PET could be considered for atypical tumour if positive at diagnosis; ENETs guidelines also support the use of 5–10 yearly bronchoscopy for follow-up if positive at diagnosis (every 1–3 years for atypical LungNETs)	Individualised decision to continue; recommended life-long (ENETS)

PanNETs, well-differentiated pancreatic neuroendocrine tumours; siNETs, well-differentiated small intestinal neuroendocrine tumours; LungNETs, well-differentiated lung carcinoids (typical/atypical); N, lymph node; T, primary tumour; CT, computerised tomography; MRI, magnetic resonance imaging; CgA, chromogranin A; SSTR, somatostatin receptor; 5-HIAA, 5-hydroxyindoleacetic acid; NSE, neuron-specific enolase; ^18^F-FDG, fluorodeoxyglucose; PET, positron-emission tomography. * not fully supported by the CommNETs/NANETS guidelines [22] with the exception of functional PanNET.
